# Van Hove singularity in the magnon spectrum of the antiferromagnetic quantum honeycomb lattice

**DOI:** 10.1038/s41467-020-20335-5

**Published:** 2021-01-08

**Authors:** G. Sala, M. B. Stone, Binod K. Rai, A. F. May, Pontus Laurell, V. O. Garlea, N. P. Butch, M. D. Lumsden, G. Ehlers, G. Pokharel, A. Podlesnyak, D. Mandrus, D. S. Parker, S. Okamoto, Gábor B. Halász, A. D. Christianson

**Affiliations:** 1 Spallation Neutron Source, Second Target Station, Oak Ridge National Laboratory, Oak Ridge, TN 37831 USA; 2 Neutron Scattering Division, Oak Ridge National Laboratory, Oak Ridge, TN 37831 USA; 3 Materials Science & Technology Division, Oak Ridge National Laboratory, Oak Ridge, TN 37831 USA; 4 Center for Nanophase Materials Sciences, Oak Ridge National Laboratory, Oak Ridge, TN 37831 USA; 5 NIST Center for Neutron Research, National Institute of Standards and Technology, Gaithersburg, MD 20899 USA; 6 Neutron Technologies Division, Oak Ridge National Laboratory, Oak Ridge, TN 37831 USA; 7 Department of Physics & Astronomy, University of Tennessee, Knoxville, TN 37996 USA; 8 Department of Materials Science & Engineering, University of Tennessee, Knoxville, TN 37996 USA

**Keywords:** Magnetic properties and materials, Quantum fluids and solids

## Abstract

In quantum magnets, magnetic moments fluctuate heavily and are strongly entangled with each other, a fundamental distinction from classical magnetism. Here, with inelastic neutron scattering measurements, we probe the spin correlations of the honeycomb lattice quantum magnet YbCl_3_. A linear spin wave theory with a single Heisenberg interaction on the honeycomb lattice, including both transverse and longitudinal channels of the neutron response, reproduces all of the key features in the spectrum. In particular, we identify a Van Hove singularity, a clearly observable sharp feature within a continuum response. The demonstration of such a Van Hove singularity in a two-magnon continuum is important as a confirmation of broadly held notions of continua in quantum magnetism and additionally because analogous features in two-spinon continua could be used to distinguish quantum spin liquids from merely disordered systems. These results establish YbCl_3_ as a benchmark material for quantum magnetism on the honeycomb lattice.

## Abstract (web summary)

Honeycomb lattices with interacting spins can host rich magnetic behaviour; however, typically features are complicated by additional interactions. Here, the authors perform neutron scattering on YbCl_3_, which exhibits near perfect two-dimensional magnetism, providing a benchmark for other materials.

## Introduction

The honeycomb lattice decorated with interacting spins is a particularly fascinating structural motif for the generation of collective quantum behavior. This bipartite lattice geometry has the minimum coordination number of three for a lattice in two dimensions. When the interactions between the spins are strongly anisotropic, as is the case for a growing number of Kitaev materials^1–22^, the result is strongly frustrating interactions and, hence, the honeycomb lattice is presently thought of as one of the primary contenders to host quantum spin liquids. In the opposite limit of isotropic spin interactions, frustrated quantum magnetism can arise through the competition of nearest neighbor and next-nearest neighbor interactions^23–37^. Indeed, most honeycomb lattice materials studied thus far require the addition of further neighbor interactions to explain the underlying physical behavior^6,8,38–47^. Such materials, with a complicated phase diagram as a function of first, second, and third nearest-neighbor interactions, have been fertile ground for exploration.

On the other hand, finding an example of honeycomb lattice magnetism where nearest-neighbor Heisenberg interactions are dominant would provide an important benchmark for studying quantum magnetism in two dimensions. In this instance, due to the bipartite geometry of the honeycomb lattice, the Heisenberg exchange interactions are not frustrated and a Néel ground state is expected^25,26,48^ at zero temperature. However, long-range order at finite temperature is prohibited by the Mermin–Wagner theorem as long as there are no anisotropic or interlayer interactions. Despite the lack of frustration, in this case, the low connectivity of the honeycomb lattice indicates that collective quantum effects are likely to be observable. Experimental realizations of the ideal honeycomb lattice Heisenberg model (HLHM) are thus attractive as a means of testing fundamental concepts of collective quantum behavior.

For example, quantum effects connected to two-magnon continua, such as magnon decays and renormalizations, have been predicted for a range of two-dimensional lattices^49^, including the square^50,51^, triangular^52,53^, and honeycomb^54^ lattices. However, while the Van Hove singularities of the two-magnon continua have a crucial role in these quantum effects, the Van Hove singularities themselves have not been experimentally observed despite extensive studies of square lattice^55–57^ and triangular lattice^58–60^ quantum magnets. More generally, it is important to distinguish quantum effects that arise entirely due to the honeycomb geometry from those that also require other sources, such as frustration or anisotropic interactions. A further motivation is the identification of a model system where the energy scale of the spin–spin interactions is modest enough to allow the quantum properties to be studied as a function of a relevant tuning parameter, for example, an applied magnetic field^54^.

**Fig. 1:**
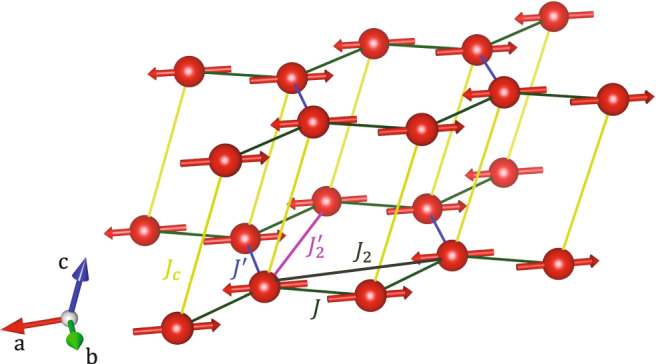
Crystal and magnetic structure of YbCl_3_. The monoclinic crystal structure (space group *C*12/*m*1) and ordered spin configuration (magnetic space group $C2^{\prime} /m$) of YbCl_3_. The monoclinic structure with *a* = 6.729 Å, *b* = 11.614 Å, *c* = 6.313 Å, and *β* = 110.6° (at *T* = 10 K) contains nearly ideal honeycomb lattices of Yb^3+^ ions (red spheres)^61^. Within each honeycomb lattice, the Yb^3+^ sites have nearest-neighbor distances of 3.884 Å and 3.867 Å for the exchanges *J* and $J^{\prime}$, respectively, and next-nearest-neighbor distances of 6.729 Å and 6.711 Å for the exchanges *J*_2_ and $J_2^{\prime}$, respectively. The resulting three bond angles for the honeycomb lattice are 120°, 119.97°, and 119.97°. The distance between the honeycomb planes is 6.313 Å, corresponding to an interlayer exchange *J*_*c*_. For the ideal model (see Eq. (1)), we consider $J=J^{\prime}$ and ${J}_{2}=J_2^{\prime} ={J}_{c}=0$. The spins are antiferromagnetically aligned within each honeycomb plane and point primarily along the *a* axis with a small tilt of 5(3)° along the *c* axis, as described in the text.

Here we focus on the nearly ideal honeycomb lattice material YbCl_3_ as a potential model quantum magnet in the unfustrated limit. The arrangement of the Yb^3+^ ions is illustrated in Fig. 1. While YbCl_3_ is formally monoclinic (space group *C*12/*m*1), there is only a very modest distortion (<0.5% difference between Yb–Yb nearest-neighbor distances) from the ideal honeycomb lattice geometry in the *ab* planes^61^. YbCl_3_ has been proposed as a candidate for Kitaev physics^62,63^, but other studies suggest that YbCl_3_ is likely to exist in the Heisenberg limit^64^. Thus, a key question concerning the physical behavior of YbCl_3_ is the nature of the spin interactions and the manifestation of collective quantum effects. Experimental studies thus far have found a broad signature in the specific heat peaked at 1.8 K that comprises  ~99.8% of the entropy of $R\mathrm{ln}\,(2)$ expected for the ground state doublet^62^. At *T* = 0.6 K, a weak anomaly in the specific heat is observed. The local crystallographic environment results in easy plane anisotropy of the Yb^3+^ magnetic moments^61^. Finally, the polycrystalline averaged magnetic excitation spectrum of YbCl_3_^61^ is rather different from that of the prototype Kitaev material RuCl_3_^17^, suggesting that a different set of interactions govern the physical behavior of YbCl_3_.

In this paper, we study YbCl_3_ with high-resolution inelastic neutron scattering (INS) measurements of single crystals. In addition to a conventional spin-wave (single-magnon) mode, these measurements show a sharp Van Hove singularity (VHS) within a broad two-magnon continuum that originates from longitudinal (quantum) spin fluctuations. Linear spin-wave theory with a single Heisenberg interaction on the honeycomb lattice reproduces all features of the data, demonstrating the strong quantum and almost perfectly two-dimensional character of YbCl_3_. Additional support for these conclusions is presented through polarized neutron diffraction and specific heat capacity measurements in conjunction with microcanonical thermal pure quantum state (mTPQ) calculations. Together, these results demonstrate that YbCl_3_ is an ideal model system of a two-dimensional quantum magnet without frustrated or anisotropic interactions. Being considerably hard to find, such model systems are crucial in quantum magnetism as they enable the controlled experimental investigation of collective quantum behavior. Moreover, on a conceptual level, the observation of a sharp feature within a continuum response demonstrates the coherent origin of the continuum. Therefore, our results for the two-magnon continuum are proof of the principle that similar features within two-spinon continua could be utilized for more unambiguous detection of fractionalized quantum magnets, such as quantum spin liquids.

## Results

### Inelastic neutron scattering data

**Fig. 2:**
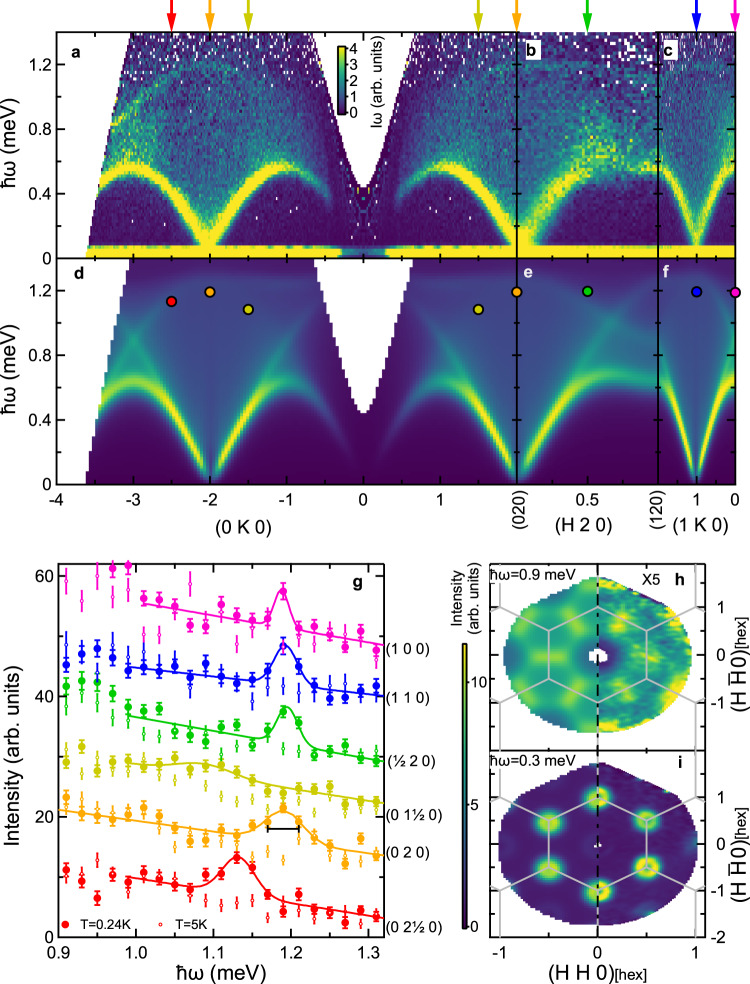
Inelastic neutron scattering data and linear spin-wave theory calculations. **a**–**c** Inelastic neutron scattering data measured at *T* = 0.24 K using the CNCS instrument. **d**–**f** Linear spin-wave calculations including transverse and longitudinal channels for the ideal model (see Eq. (1)) with a fitted value of *J* = 0.421(5) meV along high symmetry directions in the (HK0) plane. Panels **a**–**f** are plotted as the product of intensity and energy transfer (ℏ*ω*). The Yb^3+^ magnetic form factor is included in the calculations. **g** Intensity as a function of ℏ*ω* through the Van Hove singularities at several wave vectors for *T* = 0.24 K (*T* = 5 K), marked by solid (open) points. Solid lines are Gaussian lineshapes with a sloping background. The horizontal bar represents the energy resolution at ℏ*ω* = 1.19 meV. The color of the data in (**g**) corresponds to the wave vector indicated by the colored arrow at the top of (**a**)–(**c**). Peak positions of the Gaussian lineshapes in (**g**) are shown as solid circles in (**d**)–(**f**). **h**–**i** Calculated (left) and measured (right) scattering intensity for ℏ*ω* = 0.9 meV (**h**) and 0.3 meV (**i**). The data and calculation in (**h**) have been scaled by a factor of 5 to be on the same intensity scale as (**i**). Gray lines indicate high symmetry directions of the Brillouin zone. Wave vector transfers are shown in the reciprocal space of the monoclinic lattice in reciprocal lattice units for (**a**)–(**g**) and projected into a hexagonal lattice in (**h**)–(**i**). Data in (**a**)–(**c**) and (**g**) have been averaged about the *H* = 0 and *K*  = 0 axes. Data in (**h**) and (**i**) have been averaged about the vertical axis.

**Fig. 3:**
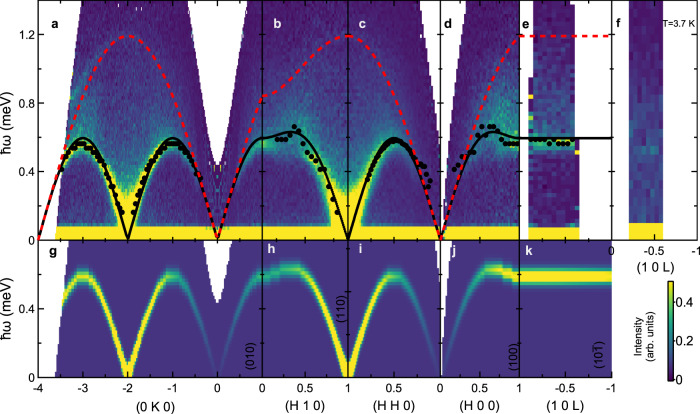
Inelastic neutron scattering data and calculations for YbCl_3_. **a**–**e** Measured at 0.24 K using the CNCS instrument. **f** Measured at 3.7 K. Black points are the locations of the absolute peak intensity at different wave vectors. The solid black line is the fitted spin-wave dispersion with a single interaction *J* = 0.421(5) meV. The dashed red line is the calculated upper boundary of the corresponding two-magnon continuum scattering. **g**–**k** Linear spin-wave calculations of the transverse spectrum for interactions *J* = 0.42(1) meV, $J^{\prime} =0.43(1)$ meV, and *J*_*c*_ = 0 meV, as described in the text. Data in (**a**)–(**f**) have been averaged about the *H* = 0 and *K* = 0 axes.

We first examine the low-energy magnetic excitation spectra of YbCl_3_ at 0.24 K. Figures 2a–c and 3a–e show the INS spectra as a function of energy, *ℏω*, and wave vector, **Q**, transfer. Figure 2a–f is plotted as the product of the intensity and the energy transfer to emphasize higher energy features in the spectrum. The spectra contain three distinct features: a component characteristic of conventional transverse spin waves (*ℏω* ≤ 0.6 meV), a continuum or multimagnon component, and a sharper component at higher energies (0.8 ≤ *ℏω* ≤ 1.2 meV). The spin-wave mode disperses throughout the (*HK*0) plane with no discernible dispersion along the (00*L*) direction (see Fig. 3e and Fig. 5 of the Supplementary material). The lack of an observable dispersion along the (00*L*) direction indicates that the interactions between the honeycomb planes are very weak. Interestingly, the spin-wave mode appears to be broader than the instrumental resolution. While the reason for this broadening is uncertain, it may arise due to domain formation as a result of bond disorder^65^, interaction with the multimagnon continuum (potentially following a renormalization of the spin-wave spectrum^52^), or even the inability to fully distinguish the overlapping continuum contribution. We also point out that the *T* = 3.7 K data in Fig. 3f illustrate a complete lack of well-formed magnetic excitations at higher temperatures. Finally, an important feature of the data is the lack of an appreciable spin gap (see Supplementary material for additional details). This observation suggests that the spins do not possess a significant uniaxial anisotropy, in agreement with the crystal field ground state with easy plane anisotropy determined in ref. ^61^.

The most unusual part of the spin excitation spectrum is the sharp feature toward the top of the broad continuum. While there is precedence for the observation of spinon and multimagnon continua in one-dimensional^66–70^ and two-dimensional square and triangular lattice^55–60^ quantum magnets, the observation of a sharp feature within such a continuum has, to the best of our knowledge, not yet been reported. This sharp multimagnon feature is explored further through constant wave vector scans in Fig. 2g. At energies where the sharp feature is well separated from the most intense parts of the continuum, the width is essentially limited to the calculated energy resolution of the instrument, FWHM = 0.04 meV at *ℏω* = 1.19 meV. As will be described in more detail below, this result provides an experimental demonstration of how a sharp feature can be generated within a continuum response in a quantum magnet. Note that the hexagonal symmetry of the spin excitations is manifest in both these higher energy features and the transverse spin-wave modes at lower energies, as shown in the right sides of Fig. 2h and i, respectively.

### Linear spin-wave theory

To understand the physics begetting the novel spin excitation spectrum of YbCl_3_, we consider a Heisenberg model on the honeycomb lattice with a single antiferromagnetic exchange interaction *J* between nearest-neighbor *S* =  1/2 spins

1 $$H=J\sum _{\langle {\bf{r}},{\bf{r}}^{\prime} \rangle }{\overrightarrow{S}}_{{\bf{r}}}\cdot {\overrightarrow{S}}_{{\bf{r}}^{\prime} }=J\sum _{\langle {\bf{r}},{\bf{r}}^{\prime} \rangle }\left[{S}_{{\bf{r}}}^{z}{S}_{{\bf{r}}^{\prime} }^{z}+\frac{1}{2}\left({S}_{{\bf{r}}}^{+}{S}_{{\bf{r}}^{\prime} }^{-}+{S}_{{\bf{r}}}^{-}{S}_{{\bf{r}}^{\prime} }^{+}\right)\right].$$

On the bipartite honeycomb lattice, the ground state $\left|0\right\rangle$ of this Heisenberg Hamiltonian *H* is the antiferromagnetic Néel state^25,26,48^. Assuming without loss of generality that the spins are parallel to the *z* direction, the transverse and the longitudinal components of the dynamical spin structure factor are

2 $${{\mathcal{S}}}_{\pm }({\bf{q}},\omega )=	 \, \frac{1}{4\pi N}\sum _{{\bf{r}},{\bf{r}}^{\prime} }\int_{-\infty }^{+\infty }\mathrm{dt}\ {e}^{i\omega t-i{\bf{q}}\cdot ({\bf{r}}^{\prime} -{\bf{r}})}\\ 	\,\left[{g}_{x}^{2}\langle 0| {S}_{{\bf{r}}^{\prime} }^{x}(t){S}_{{\bf{r}}}^{x}(0)| 0\rangle +{g}_{y}^{2}\langle 0| {S}_{{\bf{r}}^{\prime} }^{y}(t){S}_{{\bf{r}}}^{y}(0)| 0\rangle \right],\\ {{\mathcal{S}}}_{zz}({\bf{q}},\omega )=	 \, \frac{{g}_{z}^{2}}{4\pi N}\sum _{{\bf{r}},{\bf{r}}^{\prime} }\int_{-\infty }^{+\infty }\mathrm{dt}\ {e}^{i\omega t-i{\bf{q}}\cdot ({\bf{r}}^{\prime} -{\bf{r}})}\langle 0| {S}_{{\bf{r}}^{\prime} }^{z}(t){S}_{{\bf{r}}}^{z}(0)| 0\rangle ,$$

respectively, where *g*_*x*,*y*,*z*_ are appropriate *g* factors. In linear spin-wave theory, the Hamiltonian in Eq. (1) is expanded up to quadratic order in Holstein–Primakoff bosons to obtain an analytically tractable approximation (see Supplementary material for additional details). The dynamical spin structure factors in Eq. (2) are then computed by expanding the spins up to the lowest nontrivial order in the same Holstein–Primakoff bosons, which can be identified as magnon excitations. For the transverse component, expansion of the spins up to linear order gives rise to a sharp single-magnon (spin wave) contribution

3 $${{\mathcal{S}}}_{\pm }({\bf{q}},\omega )=\frac{({g}_{x}^{2}+{g}_{y}^{2})(1-| {\lambda }_{{\bf{q}}}| \cos {\vartheta }_{{\bf{q}}})}{4\sqrt{1-| {\lambda }_{{\bf{q}}}{| }^{2}}}\ \delta (\omega -{\varepsilon }_{{\bf{q}}}),$$

corresponding to the spin-wave dispersion

4 $$\begin{array}{l}\omega ={\varepsilon }_{{\bf{q}}}=\frac{3J}{2}\sqrt{1-| {\lambda }_{{\bf{q}}}{| }^{2}},\quad {\lambda }_{{\bf{q}}}=\frac{1}{3}\mathop{\sum }\limits_{j=1}^{3}{e}^{i{\bf{q}}\cdot {{\bf{r}}}_{j}},\end{array}$$

where ${e}^{i{\vartheta }_{{\bf{q}}}}={\lambda }_{{\bf{q}}}/| {\lambda }_{{\bf{q}}}|$, and **r**_1,2,3_ are the three bond vectors connecting nearest-neighbor sites on the honeycomb lattice. For the longitudinal component, the spins must be expanded up to quadratic order to get a nontrivial inelastic contribution

5 $${{\mathcal{S}}}_{zz}({\bf{q}},\omega )=\frac{{g}_{z}^{2}}{4N}\sum _{{\bf{k}}} \frac{1-\sqrt{1-| {\lambda }_{{\bf{k}}}{| }^{2}}\sqrt{1-| {\lambda }_{{\bf{q}}-{\bf{k}}}{| }^{2}}-| {\lambda }_{{\bf{k}}}| | {\lambda }_{{\bf{q}}-{\bf{k}}}| \cos ({\vartheta }_{{\bf{k}}}+{\vartheta }_{{\bf{q}}-{\bf{k}}})}{\sqrt{1-| {\lambda }_{{\bf{k}}}{| }^{2}}\sqrt{1-| {\lambda }_{{\bf{q}}-{\bf{k}}}{| }^{2}}}\ \delta (\omega -{\varepsilon }_{{\bf{k}}}-{\varepsilon }_{{\bf{q}}-{\bf{k}}}).$$

This two-magnon contribution gives a broad continuum over a finite energy range for each momentum **q** because the energy transfer, *ω* = *ε*_**k**_ + *ε*_**q**−**k**_, depends on the momenta **k** and **q** − **k** of the individual magnons. We note that, in linear spin-wave theory, the staggered magnetic moment of the Néel state is only  ≈ 48% of its classical value on the honeycomb lattice, in comparison to  ≈ 61% on the square lattice^71^. Such a large reduction of the magnetic moment indicates that quantum fluctuations are strong due to the low coordination number of the honeycomb lattice^72^.

## Discussion

### Comparison between data and model

The HLHM in Eq. (1) reproduces the experimental data for the transverse spin-wave mode, the broad continuum, and the sharp feature toward the top of the continuum. We first note that, due to the summation over the momentum **k**, the contribution from the two-magnon states in Eq. (5) results in a broad continuum of scattering. To determine the Heisenberg exchange *J*, we consider the transverse component of the data. Due to the large scattering intensity of the continuum, we compare the calculated dispersion to the overall maxima in the scattering intensity as a function of **q** (solid points in Fig. 3a–e). Comparing these values for points restricted to the (*HK*0) plane yields a Heisenberg exchange of *J* = 0.421(5) meV, shown as a solid line in Fig. 3a–e. Moreover, we directly compare the data along the (0*K*0), (*H*20), and (1*K*0) directions to the numerical evaluation of Eqs. (3) and (5) convolved with a Gaussian approximation to the instrumental energy and wave vector resolution functions while also including the spherical approximation for the Yb^3+^ magnetic form factor and an additive background term. The resulting spectra are shown in Fig. 2d–f and h–i; the continuum response and the sharp feature within the continuum are reproduced exceptionally well. (see  Supplementary material for additional comparisons between the data and the model.) We remark that the excellent agreement between the experimental data and the ideal HLHM is also in accordance with the prediction of ref. ^64^.

**Fig. 4:**
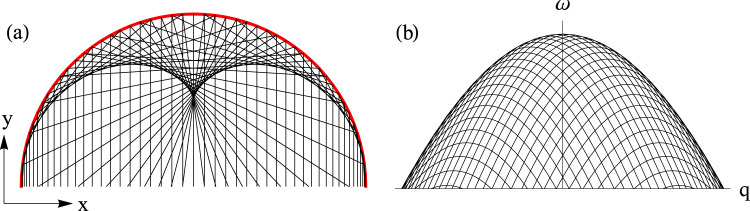
Alternative understanding of Van Hove singularities as caustic features in the collective spin dynamics. **a** Caustic features in ray optics. Parallel light rays (black lines) enter an optical system at different positions. When these light rays reflecting from a circular mirror (red line) coalesce, they give rise to caustic features in real space. **b** Caustic features in a spin response. The two-magnon continuum can be understood as a sum of sharp contributions, *ω* = *ε*_**k**_ + *ε*_**q**−**k**_, each corresponding to a fixed momentum **k** of the first magnon. When these sharp contributions (black lines) coalesce, they give rise to caustic features in the two-magnon continuum. Note that the spin response shown here is for a one-dimensional model system; for the two-dimensional system in consideration, the caustic features appear inside the continuum (not at its edge) and are weaker as they correspond to logarithmic (rather than square-root) singularities.

The sharp feature toward the top of the continuum is a particularly interesting aspect of the spectrum that, to the best of our knowledge, has not been previously observed in a quantum magnet. In the model, such a sharp feature appears within the two-magnon continuum due to a VHS in the joint density of states^49,50,52–54^. Indeed, on the level of pure kinematics (i.e., ignoring any matrix element effects), the longitudinal two-magnon response in Eq. (5) is proportional to the joint density of states, ${\hat{g}}_{{\bf{q}}}(\omega )={\sum }_{{\bf{k}}}\delta (\omega -{\varepsilon }_{{\bf{k}}}-{\varepsilon }_{{\bf{q}}-{\bf{k}}})$, at each momentum **q**, which corresponds to the joint band dispersion ${\hat{\varepsilon }}_{{\bf{q}}}({\bf{k}})={\varepsilon }_{{\bf{k}}}+{\varepsilon }_{{\bf{q}}-{\bf{k}}}$ as a function of the individual magnon momentum **k**. Being a two-dimensional band dispersion, ${\hat{\varepsilon }}_{{\bf{q}}}({\bf{k}})$ has a saddle-point VHS which gives a logarithmic divergence in the density of states ${\hat{g}}_{{\bf{q}}}(\omega )$ and, thus, in the longitudinal spin response. Physically, the VHS is a specific energy transfer *ω* that can create many distinct magnon pairs with a fixed total momentum **q** but different individual momenta **k** and **q** − **k**. The coalescence of such distinct scattering processes is analogous to the coalescence of light rays giving rise to caustic features in ray optics (see Fig. 4). Therefore, using this analogy, the VHS can also be understood as a caustic feature in the longitudinal spin dynamics. We emphasize that the observation of a VHS is direct evidence for strong quantum fluctuations in YbCl_3_ (because the VHS appears in the longitudinal spin response) as well as the two-dimensional nature of its quantum magnetism (because significant interlayer exchange would smear out the VHS).

Upon close examination, the analytic model does not fully capture the intensity and dispersion of the VHS over the entire zone, as can be seen in Fig. 2a–f. By plotting the fitted peak positions of the VHS from Fig. 2g on the calculated spectra in Fig. 2d–f, we notice that there are differences between the calculated and the observed VHS energies near the (100) and $(\frac{1}{2}20)$ wave vectors  (≈0.2 meV). These energy differences may arise from a renormalization of the two-magnon continuum due to magnon interactions neglected in linear spin-wave theory. Also, we point out that domain formation due to bond disorder^65^ could, in principle, play a role in partially smearing out the VHS and thus explaining why certain portions of the predicted VHS are absent from the experimental data.

We now explore the potential importance of additional exchange interactions through comparison of the experimental spin-wave dispersion, including points measured along the *L* direction, to calculations using the SpinW software^73^. Since there are very small differences in the bond lengths within the honeycomb layers of YbCl_3_, as described in Fig. 1, we label two of the three nearest-neighbor exchanges as *J* for the *d* = 3.884 Å bonds and the third one as $J^{\prime}$ for the *d* = 3.867 Å bond. The numerical comparison yields *J* = 0.42(1) meV and $J^{\prime} =0.43(1)$ meV, while *J*_*c*_ refines to a value below the detection limit of 0.016 meV for the measurements reported here (see  Supplementary material for additional details). The resulting cross-section is shown in Fig. 3g–k. The numerically determined *J* and $J^{\prime}$ are indistinguishable from each other and close to the value determined by a comparison to the analytical model. The spin-wave modes from linear spin-wave theory accurately reproduce the dispersion and intensity distribution of this portion of the measured spectrum (Fig. 3g–k). We also attempted to include next-nearest-neighbor exchange interactions within the plane of the honeycomb lattice; the best fit values of *J*_2_ and $J_2^{\prime}$ are three orders of magnitude smaller than *J* and zero within the error bars of the refined value (see Supplementary material for additional details).

### Magnetic moment and Néel temperature

To further check the validity of the ideal HLHM, we have performed polarized neutron diffraction measurements at *T* =  0.3 K (see Supplementary material for additional details). We observe an antiferromagnetic spin arrangement with a well-defined staggered magnetic moment. By symmetry, this moment can either lie in the *ac* plane or point along the *b* axis, i.e., a linear combination of the two directions is not allowed. Our measurements reveal that the magnitude of the ordered moment is 1.06(4) *μ*_*B*_ and that the moment points primarily along the *a* axis with only a small deviation of 5(3)° along the *c* axis. The expected moment from the ground state crystal field doublet is 2.24(5) *μ*_*B*_^61^. The value of the ordered moment is thus  ≈47% of the fully polarized moment, which is in excellent agreement with the value of  ≈48% obtained from linear spin-wave theory and the value of  ≈54% determined by more accurate techniques^72,74–77^. We emphasize, however, that these theoretical values are extremely sensitive to any interlayer exchange *J*_*c*_. For example, if we include ∣*J*_*c*_/*J*∣ ≈ 4%, corresponding to the detection limit from the INS response (see above), the ordered moment in linear spin wave theory already increases from  ≈48% to  ≈63%. Therefore, the small value of the experimental moment indicates that the actual value of *J*_*c*_ is significantly below the INS detection limit and further confirms our conclusion that the magnetism in YbCl_3_ is two dimensional to a very good approximation.

The same conclusion is demonstrated even more strikingly by the very small value of the Néel temperature, *T*_*N*_ ≈  0.6 K^62^. Theoretically, for a quasi-two-dimensional Heisenberg magnet, the Néel temperature is approximately given by ${T}_{N}=4\pi {\rho }_{s}/\mathrm{log}\,| J/{J}_{c}|$, where *ρ*_*s*_ is the spin stiffness of the purely two-dimensional system. At this temperature, *T* = *T*_*N*_, the individual interlayer exchanges *J*_*c*_ add constructively within an area of linear size $\xi \sim \exp (2\pi {\rho }_{s}/T)$^78,79^ to produce an effective interlayer exchange on the order of the intralayer exchange *J*. Taking *ρ*_*s*_ ≈ 0.101*J* from the literature^76,77^ and *J* ≈ 0.42 meV from the neutron scattering data, the relative interlayer exchange is then estimated to be $| {J}_{c}/J| \sim \exp (-4\pi {\rho }_{s}/{T}_{N})\approx 3\times 1{0}^{-5}$. This value is three orders of magnitude smaller than the detection limit from the INS response and indicates that the magnetism in YbCl_3_ is as close to two-dimensional as in the canonical cuprate antiferromagnet La_2_CuO_4_^80^. Moreover, the specific ordering pattern below *T*_*N*_ signals the presence of very small anisotropic interactions on top of the dominant Heisenberg exchange *J*. Since these anisotropic spin interactions should also contribute to *T*_*N*_, the interlayer exchange may be even smaller than our estimate. We finally remark that the application of a small magnetic field should, in principle, cause the ordering transition to sharpen and the Néel temperature to increase^79^. While there are experimental indications for such behavior in the specific heat^62^, more detailed studies of the field dependence would be very important to confirm not only this prediction but also further predictions of interesting quantum effects at larger magnetic fields^54^.

### Heat capacity calculations

**Fig. 5:**
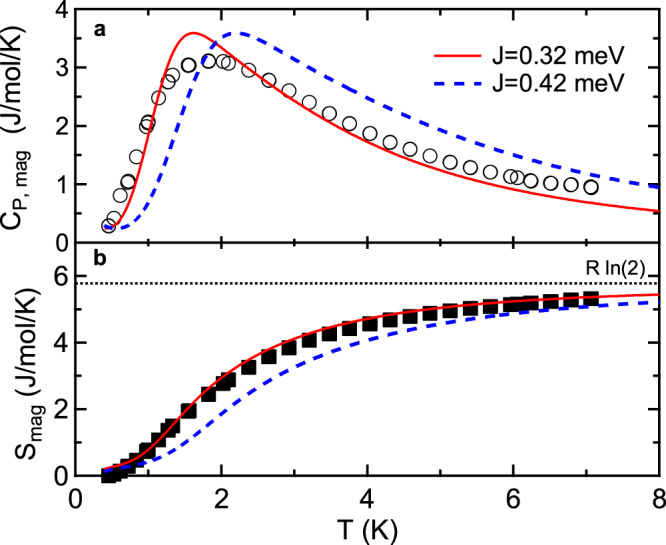
Magnetic specific heat capacity, entropy, and mTPQ calculations for YbCl_3_. Specific heat capacity (**a**) and entropy (**b**) for YbCl_3_. The solid red line is the best fit calculation using mTPQ as described in the text. The dashed blue line is the calculated specific heat using mTPQ with the value *J* = 0.42 meV obtained by fitting the INS data.

Specific heat capacity measurements provide an additional means of examining the HLHM in YbCl_3_. The experimental specific heat and the entropy of YbCl_3_ for 0.5 K < *T* < 8 K are shown in Fig. 5a and b. Between *T* = 0.5 K and *T* = 8 K, nearly all of the entropy, $R\mathrm{ln}\,(2)$, for the ground state doublet has been recovered by the system with only a very small contribution in the region of the transition to long-range magnetic order^62^. We use mTPQ calculations^81^, as implemented in the ${\mathcal{H}}\Phi$ library^82^, for a cluster size of 32 spin $\frac{1}{2}$ elements to calculate the heat capacity as a function of the reduced temperature *T*/*J* (see Supplementary material for additional details). The results for the HLHM with *J* = 0.42 meV, obtained by fitting the INS data, are shown in Fig. 5a and b. The overall shape is in reasonable agreement with the data, but a somewhat improved comparison is found by using *J* = 0.32 meV. The discrepancy may be due to quantum corrections, such as renormalizations due to magnon interactions^51,52^, that are captured by the mTPQ calculations but neglected in linear spin wave theory.

We have used INS to investigate the collective magnetic excitation spectrum of YbCl_3_. In addition to a conventional transverse spin-wave (single-magnon) mode, there is a longitudinal two-magnon continuum harboring a sharp VHS. These components are all reproduced by linear spin-wave theory with a single nearest-neighbor Heisenberg interaction on the honeycomb lattice. The sharp VHS is observable due to the almost perfectly two-dimensional quantum magnetism in YbCl_3_, which is further reflected in a strongly reduced ordered moment and a very small ordering temperature. Our results establish YbCl_3_ as an ideal model system to investigate the collective quantum behavior of the honeycomb antiferromagnet in the unfrustrated limit. Finally, we point out that the observation of a VHS in a two-magnon continuum provides a strong indication that a similar observation in the two-spinon spectrum of a two-dimensional quantum spin liquid^83,84^ is experimentally feasible. Such an observation in a quantum spin liquid would be important in ruling out competing sources of a continuum response, such as quenched disorder or overdamped magnons, and could provide a more unambiguous signature of a long-range-entangled quantum state.

## Methods

Single crystals of YbCl_3_ were grown using the Bridgman technique in evacuated silica ampoules (see Supplementary material and ref. ^85^ for further details). INS measurements were performed with the cold neutron chopper spectrometer (CNCS)^86^ at the Spallation Neutron Source at Oak Ridge National Laboratory. Additional measurements were made with the disk chopper spectrometer (DCS) at NIST (see Supplementary material for additional details). The CNCS measurements were performed with a 0.625 g sample oriented with the (*HK*0) scattering plane horizontal using 2.49 meV incident energy, *E*_*i*_, neutrons in the high flux configuration of the instrument. To minimize the effects of the modest neutron absorption cross section of Yb and Cl, the sample used at CNCS was constructed of a stack of plates cut to dimensions of 3.2 mm by 3.4 mm. The polarized HYSPEC measurements were performed using the same sample as the CNCS experiment. We employed 3D polarization analysis, i.e., we measured the *P*_*x*_, *P*_*y*_, and *P*_*z*_ spin-flip and non-spin flip channels. Additional details are provided in the Supplementary material.

## Supplementary information

Supplementary Information

Peer Review File

## Data Availability

The authors declare that the data supporting the findings of this study are available within the article and its Supplementary Information files. Additional data are available from the corresponding author upon reasonable request.

## Code availability

All numerical codes used in this paper are available from the corresponding author upon reasonable request.
