# Supplementary material for: Van Hove singularity in the magnon spectrum of the antiferromagnetic quantum honeycomb lattice
Source: Nat Commun. 2021 Jan 8;12:171. doi: 10.1038/s41467-020-20335-5 (PMC7794317; doi:10.1038/s41467-020-20335-5)
Supplement: Supplementary file 1 — Supplementary Information [file 41467_2020_20335_MOESM1_ESM.pdf]

Supplemental information for: Van Hove singularity in the magnon spectrum of the antiferromagnetic quantum honeycomb lattice

G. Sala,[1,2] M. B. Stone,[2] Binod K. Rai,[3] A. F. May,[3] Pontus Laurell,[4] V. O. Garlea,[2] N. P. Butch,[5] M. D. Lumsden,[2] G. Ehlers,[6] G. Pokharel,[7,3] A. Podlesnyak,[2] D. Mandrus,[8,3,7] D. S. Parker,[3] S. Okamoto,[3] Gábor B. Halász,[3] and A. D. Christianson[3,*]

[1]*Spallation Neutron Source, Second Target Station, Oak Ridge National Laboratory, Oak Ridge, Tennessee 37831, USA*

[2]*Neutron Scattering Division, Oak Ridge National Laboratory, Oak Ridge, Tennessee 37831, USA*

[3]*Materials Science & Technology Division, Oak Ridge National Laboratory, Oak Ridge, TN 37831, USA*

[4]*Center for Nanophase Materials Sciences, Oak Ridge National Laboratory, Oak Ridge, Tennessee 37831, USA*

[5]*NIST Center for Neutron Research, National Institute of Standards and Technology, Gaitersburg, MD 20899, USA*

[6]*Neutron Technologies Division, Oak Ridge National Laboratory, Oak Ridge, TN 37831, USA*

[7]*Department of Physics & Astronomy, University of Tennessee, Knoxville, TN 37996, USA*

[8]*Department of Materials Science & Engineering, University of Tennessee, Knoxville, TN 37996, USA*

(Dated: November 7, 2020)

* christiansad@ornl.gov

I. SAMPLE PREPARATION AND SPECIFIC HEAT MEASUREMENTS

Single crystals of $YbCl_3$ were grown using a gradient freeze technique (static Bridgman method)[1]. All handling steps were performed in a helium glove box. The polycrystalline source (Alfa Aesar product 40653) was placed in silica ampoules formed with tapered ends, which were heated to 250°C while under dynamic vacuum for approximately 24 h. The evacuated ampoules were then sealed, affixed to a silica rod for support, and hung in a vertical tube furnace. The ampoules were heated to 900°C, where the melt was homogenized for 12 h prior to cooling to 790°C at 0.4°C/hr in an intentional temperature gradient. Upon opening the ampoules in the glove box, large grains (several cm long) were identified by eye and easily cleaved apart to reveal facets with [001] normal.

For specific heat measurements, crystals were cleaved apart and cut into thin plates using a blade. Apiezon N-grease was employed to affix the sample to the measurement platform in a Quantum Design (QD) Physical Property Measurement System with a QD ^{3}He insert. In the glove box, the crystal was coated with the grease to protect the sample from the air while loading the sample into the ^{3}He insert. The grease-coated sample was exposed to air for less than 120 s prior to evacuation of the measurement chamber. A polycrystalline piece of $LuCl_3$ (Alfa Aesar 35802) was measured in a similar manner down to 1.9 K. These data were utilized for the phonon background for $YbCl_3$ above 2 K. The phonon background below 2 K is essentially negligible relative to the large magnetic specific heat of $YbCl_3$. In this temperature range, the phonon background was extrapolated using a Debye model obtained by fitting the data for $LuCl_3$ between 2 and 3 K.

Single crystal samples for neutron scattering measurements were wrapped in Cu foil to maximize thermal contact. Samples were kept sealed under He gas in copper containers to prevent contamination and decomposition by air. Samples remained in the copper containers for measurements. Due to the large neutron scattering cross-section of chlorine, additional care was taken to reduce the profile of samples that were measured in the $(HK0)$ scattering plane. These samples were prepared by carefully cutting as-grown crystals into pieces approximately 3×3 mm^2 in lateral dimensions. These individual pieces were then co-aligned by stacking along the c-axis while maintaining the in-plane orientation.

**Hamiltonian**	J	J'	J_2	J_2'	J_c	χ^2
Heisenberg I *	0.421(5)					
Heisenberg II	0.432(8)	0.430(6)				0.743
Heisenberg III	0.42(1)	0.43(1)			-3.7e^{-4}(3e^{-5})	0.752
Heisenberg III	0.435(9)	0.44(1)	-0.002(2)			0.799
Heisenberg IV	0.43(1)	0.440(7)	-0.00002(2)	-0.0005(5)		0.805

TABLE I. *Results of $YbCl_3$ dispersion refinement for different Heisenberg Hamiltonians.* Refined exchange constants for the Heisenberg Hamiltonian models described in the text. Values of exchange constants are in units of meV. χ^2 is the reduced chi square value from a Levenberg-Marquardt fit which used spinW to calculate the spin wave dispersion. The roman numerals identify how many exchange couplings were used in the Hamiltonian with respect to Fig. 1 in the main text. Heisenberg II, III, and IV models were refined using values of energy transfer for inelastic peak locations of 280 constant wave-vector scans along the (H00), (0K0), (H10), (1K0), (10L), and (HK0) directions. (*) The Heisenberg I refinement uses values of the energy transfer for inelastic peak locations of constant wave-vector scans in the (HK0) plane to refine the analytic expression for the single magnon spin-wave dispersion of the honeycomb antiferromagnet, (Eq.4) described in the main text. The Heisenberg I refinement was not performed with SpinW so its χ^2 value is not listed.

II. INELASTIC NEUTRON SCATTERING ANALYSIS

Inelastic neutron scattering measurements were performed using three instruments: the Disk Chopper Spectrometer (DCS) [2] at NIST, two series of measurements using the Cold Neutron Chopper Spectrometer (CNCS) at ORNL [3] using incident energies of 3.27 meV, 2.49 meV respectively. The measurements were performed with the sample in the (HK0) scattering plane. Temperature control for these experiments was provided by a ^{3}He cryogenic insert placed within a liquid helium cryostat.

Measurements were performed at CNCS(DCS) by rotating the sample through 200(220) degrees in 1 degree steps. Measurements at HYSPEC were performed by rotating the sample through 200 degrees in 1 degree steps with the central instrument detector (S_2) located at scattering angles of -38 and -68 degrees. Data were normalized to detector sensitivity and processed using MAN-

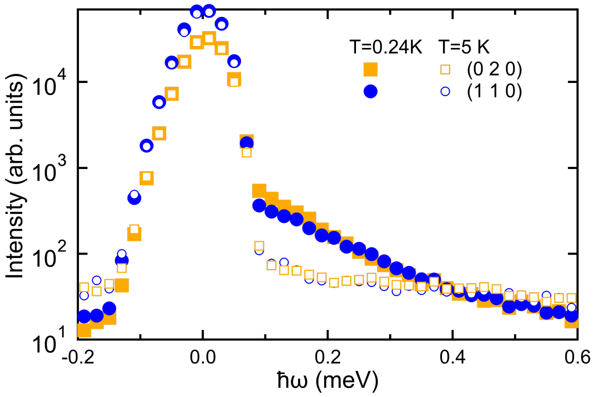

FIG. 1. Intensity as a function of $\hbar\omega$ from the CNCS measurement described in the main text. Data are shown for two reciprocal lattice points for both $T = 0.24$ K (solid points) and $T = 5$ K (open points). Data were integrated over ±0.1 rlu for all three directions in reciprocal space.

TID [4] to be in units proportional to the dynamic structure factor. Except where indicated, the range of L used for integration of the CNCS data was from $L = -1.3$ to $L = 1.3$ rlu. The range of integration used for data orthogonal to slices shown is ±0.1 rlu. The range of integration used for constant wave vector scans shown is ±0.1 rlu in each direction.

For the configurations of the instruments used the calculated energy resolution at $\hbar\omega = 0$ is $\delta\hbar\omega = 100\ \mu$eV and $\delta\hbar\omega = 74\ \mu$eV at DCS and CNCS respectively. Data were averaged across the highest symmetry directions of the Brillouin zone by applying the proper Laue symmetry operations of the $C2/m$ space group. In particular, each symmetry operation was applied to the calculated UB matrix of the sample in order to generate the symmetrized data. Then, all the symmetrized data were merged together and normalised by proton charge to maximize use of the statistical content of each data set. Note that symmetry operations which project the vertical plane into the scattering plane were removed to avoid the possible creation of artifacts.

The SpinW fitting of the dispersion relation was performed in two steps: first using the *Matlab* particle swarm optimizer algorithm, iterating the process 2500 times to decrease statistical noise to find starting parameters. Second combining spinW and the Levenberg-Marquardt lmfit library to obtain more reliable values and related error bars. Table I shows the results of this comparison adding different exchange correlations between first, second and third nearest neighbors. This includes neighbors along the c-axis as shown in Fig. 1 in the main text. Refinement of the exchange parameters was performed based upon scans along the $(H00)$, $(0K0)$, $(H10)$, $(1K0)$, $(10L)$, and $(HH0)$ directions using a total of 280 wave-vectors. The refined J_2 and J_2' exchange terms corre-

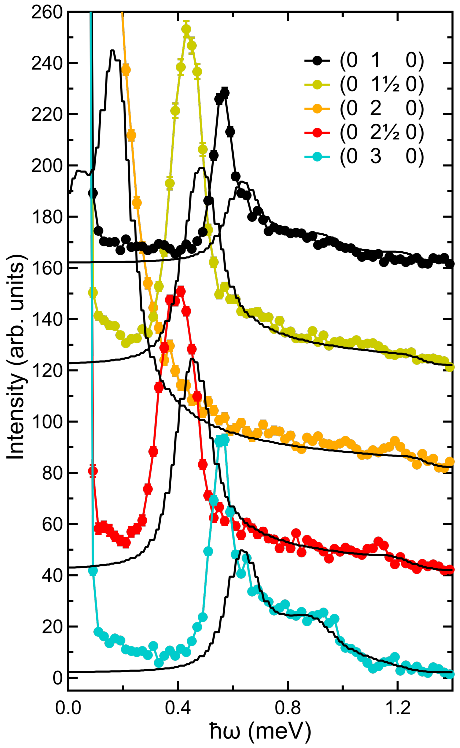

FIG. 2. Intensity as a function of $\hbar\omega$ from the CNCS measurement described in the main text. Data are shown for $T = 0.24$ K for wave-vectors along the $(0K0)$ direction. Data were integrated over ±0.1 rlu for all three directions in reciprocal space. Solid lines correspond to fits to the ideal HLHM model including longitudinal and transverse excitations as described in the main text.

spond to next-nearest neighbor exchange within the honeycomb layer as shown in Fig. 1 of the main text. The refined values are statistically equivalent to zero and do not improve the refinement. The dispersion values refined in SpinW were determined from the absolute peak location in the scattering intensity. This was done because refining a dispersion based upon Gaussian lineshapes fitted to each constant wave-vector scan skewed the dispersion to higher energy transfers because of the large continuum scattering contribution to the cross-section. The error in energy transfer was chosen to be the width associated with ±10% of maximum scattering intensity for each respective constant wave-vector scan. SpinW minimizes the goodness of fit parameter R_w to refine the dispersion. SpinW was not used to refine the single J HLHM model where $J = J'$; however, the refinement of the $J \neq J'$ model yields statistically equivalent exchange values as shown in Tab. I for the Heisenberg II Hamiltonian

III. INELASTIC NEUTRON SCATTERING DATA

Figure 1 examines constant wave-vector scans in the vicinity of antiferromagnetic zone centers. Data are shown for $T = 0.24$ K and $T = 5$ K. Any potential gap in the excitation spectrum would be for energies below approximately 0.1 meV.

Figures 2-4 are constant wave-vector scans through the $T = 0.24$ K CNCS measurement along the $(0K0)$, $(1H0)$, and $(H20)$ directions respectively. Many of these scans appear in the main text over a narrow range of energy transfer in Fig. 1(g). The solid lines correspond to the refinement of the measured spectrum compared to the ideal HLHM model including longitudinal and transverse excitations as described in the main text.

Figure 5 shows cuts along $\hbar\omega$ at the $(10L)$ and $(0.9\ 0.9\ L)$ reciprocal lattice positions for several values of L. No dispersion along L is discernible from the data. To be observable, a dispersion along L would have to deviate by the HWHM of the peak width which is 0.0308 meV for the $(10L)$ peaks and 0.066 meV for the $(0.9\ 0.9\ L)$ peaks respectively. Using this criterion, the experimental detection limit was determined by examining how the calculated dispersion deviates from $J_c = 0$ for finite values of J_c. With this procedure, the detection limit is found to be 0.016 meV and 0.012 meV for the $(10L)$ and $(0.9\ 0.9\ L)$ peaks respectively. As indicated in the main text, we adopt the more conservative 0.016 meV as the detection limit of the experiment. Attempts to refine a value of J_c resulted in values below both of the detection limits listed above.

Measurements were also performed at the DCS instrument at the NIST Center for Neutron research. These data are shown in Fig. 6(a)-(c) for $T = 12$ K, $T = 0.24$ K and the difference of the low temperature and high temperature data respectively. These results are shown as a powder average of the single crystal measurement performed at the DCS instrument. The difference data provide a reliable indication of the lower bounds of the magnetic spectrum.

IV. POLARIZED NEUTRON DIFFRACTION

The ordered spin configuration was examined using polarized neutron diffraction with the HYSPEC instrument[5] at the Spallation Neutron Source at Oak Ridge National Laboratory. The measurements using polarised neutrons ($E_i = 3.8$ meV) were performed with the same sample used at CNCS, and aligned in the $(HK0)$ scattering plane. Temperature control for this experiments was provided by a ^{3}He cryogenic insert placed within a liquid helium cryostat.

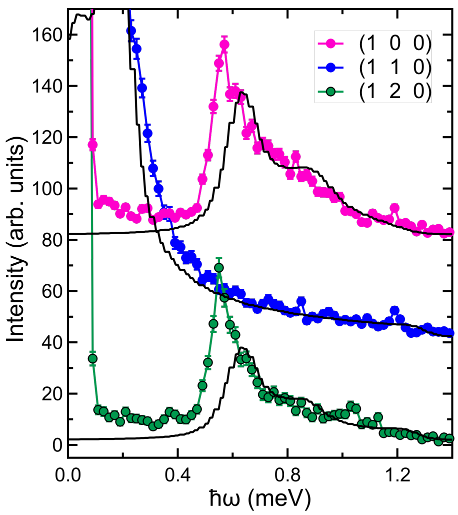

FIG. 3. Intensity as a function of $\hbar\omega$ from the CNCS measurement described in the main text. Data are shown for $T = 0.24$ K for wave-vectors along the $(1K0)$ direction. Data were integrated over ±0.1 rlu for all three directions in reciprocal space. Solid lines correspond to fits to the ideal HLHM model including longitudinal and transverse excitations as described in the main text.

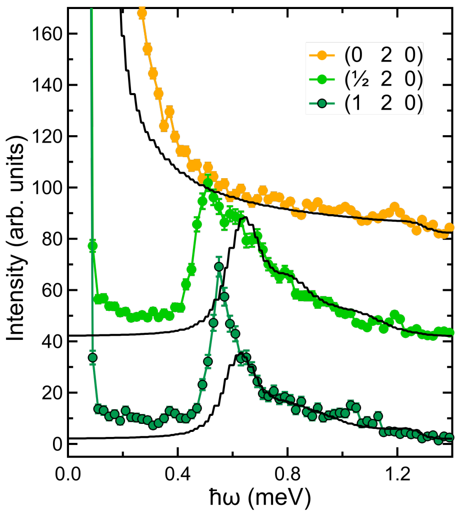

FIG. 4. Intensity as a function of $\hbar\omega$ from the CNCS measurement described in the main text. Data are shown for $T = 0.24$ K for wave-vectors along the $(H20)$ direction. Data were integrated over ±0.1 rlu for all three directions in reciprocal space. Solid lines correspond to fits to the ideal HLHM model including longitudinal and transverse excitations as described in the main text.

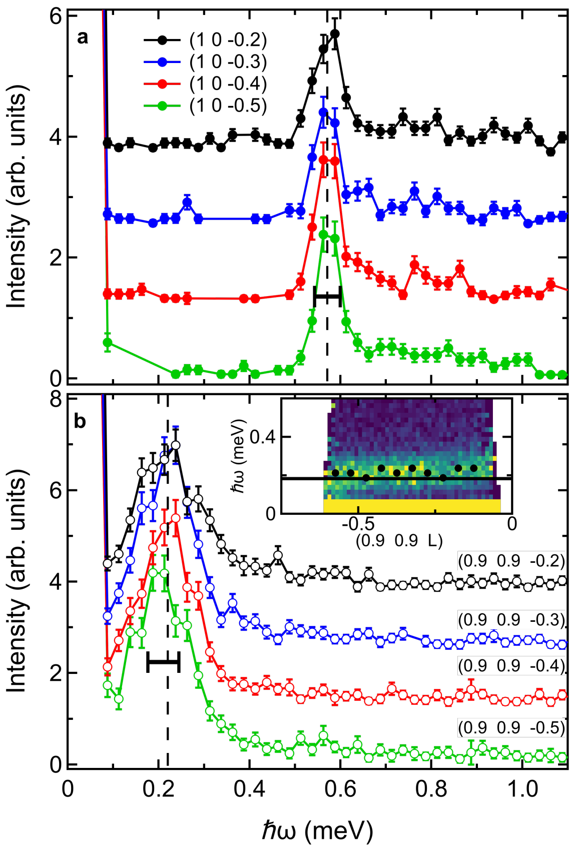

FIG. 5. Intensity as a function of $\hbar\omega$ from the second CNCS measurement. Wave-vector transfers listed are shown in the monoclinic setting of the crystal. The measurement has been integrated along the H and K wave-vectors by 1 ± 0.05 and 0 ± 0.05 rlu (reciprocal lattice units) respectively. The L integration range is the listed value in the legend ±0.025 rlu. The dashed vertical line corresponds to the average peak energy of the constant wave-vector scans shown in the figure. The horizontal bar at the midpoint of the (1 0 − 0.5) data is the FWHM instrumental energy resolution of the spectrometer at 0.57 meV energy transfer.

The crystal ensemble was rotated through 120 degrees in 1 degree steps to cover the (020) and (110) Bragg peaks, with the central instrument detector (S_2) located at a scattering angles of −55 degrees. Data were normalized to detector sensitivity using TiZr and processed using MANTID [4] to account for beam deflection angle (1.8 degrees) due to the super-mirror array, proton charge normalisation, and flipping ratio. The flipping ratio for the experiment was determined from the measurement of a TiZr standard and found to be 13. This flipping ratio was used to determine the six components of the polarization tensor considered here. The flipping ratio corrections were done according to the procedure described in Ref. [6].

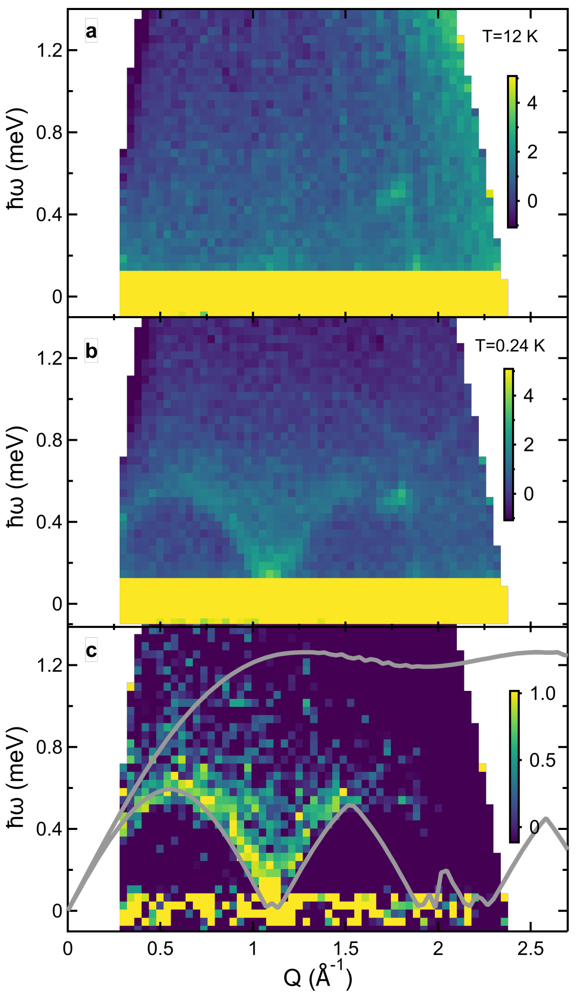

FIG. 6. Dynamic structure factor $S(Q, \hbar\omega)$ for $YbCl_3$ measured at DCS at $E_i = 3.27$ meV. To improve counting statistics the single crystal measurement has been powder averaged for all orientations of the sample measured at the spectrometer. (a) Data measured at $T = 12$ K. (b) Data measured at $T = 0.24$ K. (c) High temperature data subtracted from the low temperature data. The solid grey lines are the calculated bounds of the powder averaged spectrum for the value $J = 0.42$ meV. The lower bound is determined from the analytic expression for the dispersion of the ideal HLHM described in the main text. The upper bound is determined from two-magnon excitations out of the transverse spin-wave mode.

Within this configuration, we mapped all the six components of the polarisation tensor along P_x , P_y and P_z (polarization || **Q**, ⊥ **Q** in the the horizontal plane, and ⊥ **Q** out of the horizontal plane respectively) for both spin flip (SF) and non-spin flip (NSF). The range of L used for the out of plane integration was $L = \pm 1.3$ rlu. The range of integration used for data orthogonal to slices shown is ±0.1 rlu. The range of integration used for constant wave vector scans shown is ±0.1 rlu

in each direction.

We now discuss the formalism used to extrapolate the magnitude and direction of the magnetic moment of $YbCl_3$, based on our polarized neutron diffraction measurements. First, we note that symmetry considerations based on the space group of this system ($C12/m1$) indicate that the magnetic moment is constrained to either point along the b-axis or lie in the ac-plane. In particular, if the magnetic moment is parallel to the b-axis then a neutron scattering measurement of the (020) peak would not result in any magnetic intensity. This clearly is not the case as shown in Fig. 7, and hence we can already conclude that the moment is in the ac-plane and that the magnetic space group is $C2'/m$ (#12.60).

With the aforementioned constraints, the six components of the polarisation tensor along P_x , P_y and P_z (polarization || **Q**, ⊥ **Q** in the the horizontal plane, and ⊥ **Q** out of the horizontal plane respectively) for both spin flip (SF) and non-spin flip (NSF), provide the following information[6, 7]:

$$\begin{cases} I_{SF_x} \propto (Ma^* + Mc) & I_{NSF_x} \propto N \\ I_{SF_y} \propto Mc & I_{NSF_y} \propto Ma^* + N \\ I_{SF_z} \propto Ma^* & I_{NSF_z} \propto Mc + N \end{cases} \tag{1}$$

where M and N are the magnetic and nuclear structure factors respectively.

To understand the results of the measured polarized neutron scattering cross-sections, we consider the projection of the magnetic moment components along the axes as shown in the inset of Fig. 7. Within this reference system, we have:

$$\begin{cases} ma^* = m\sin(\beta - \psi) \\ ma = m\sin(\beta - \psi)/\sin(\beta) \\ mc = m\sin(\beta - \psi)[\cot(\beta - \psi) - \cot(\beta)] \end{cases} \tag{2}$$

where m is the magnetic moment of Yb spins, the monoclinic angle $\beta = 110.55°$ and ψ is the tilt angle of the spins calculated from the a-axis. It is immediately evident from Eqs. 1 and 2 that, if $I_{SF_x} = I_{SF_z}$ and $I_{SF_y} = 0$, then $mc = 0$, thus $\psi = 0$.

To determine the integrated intensities, Gaussian curves constrained to the same width were fitted to the data for the six polarisation channels with the corrections applied as indicated above. In the present case, $I_{SF_y} \neq 0$, therefore we can estimate the ψ angle from the ratio of I_{SF_y}/I_{SF_z}, and

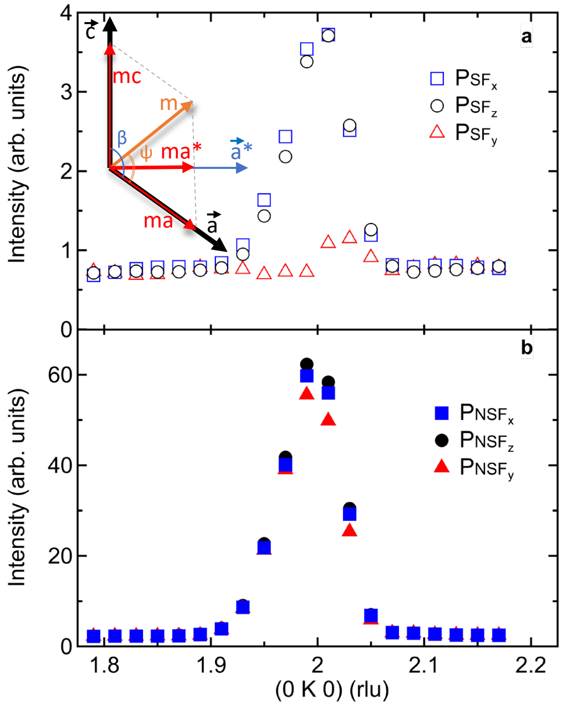

FIG. 7. Results of the fit of the HYSPEC polarisation data set collected with $T = 0.3$ K for the SF (a) and NSF (b) channels. The inset shows the reference coordinate system we adopted for the calculation of the Yb magnetic moment, and the tilt angle ψ with respect to the monoclinic a-axis.

the magnetic moment from the ratio I_{NSF_x}/I_{SF_y}. Based on our fit:

$$\frac{I_{SF_y}}{I_{SF_z}} = \frac{Mc}{Ma^*} = \cot(\beta - \psi) - \cot(\beta) = 0.105(6) \qquad (3)$$
$$\rightarrow \psi = 5(3)^\circ$$

$$\frac{I_{NSF_x}}{I_{SF_x}} = \frac{N}{(Mc + Ma^*)} = \frac{N}{M\sin(\beta - \psi)[1 + \cot(\beta - \psi) - \cot(\beta)]} \qquad (4)$$
$$\rightarrow m = 1.07(4)\mu_B$$

Note the normalization value $N/M = 1.143$ was estimated using the Fullprof software. Our spin wave theory predicts that the magnetic moment of Yb^{3+} would be reduced by 48% from the classical value. Indeed our previous results determined that a magnetic moment of the ground state doublet of $m = 2.25\mu_B$/Yb, hence we have: $m = 2.25 * 0.48 = 1.08\mu_B$/Yb, which is in excellent agreement with the polarized neutron diffraction results described here.

In summary, the Yb spins are preferentially pointing along the monoclinic a-axis, with a small tilt (5(3)°) along the c-axis) and a magnitude $m = 1.07(4)\ \mu_B$. A magnetic .cif file corresponding

to this structure created using resources at the Bilbao Crystallographic Server[8] is provided as separate file. We note that the magnetic structure determined here is largely in agreement with that determined in Ref. [9].

V. LINEAR SPIN-WAVE THEORY OF THE DYNAMICAL SPIN STRUCTURE FACTOR

We consider a nearest-neighbor antiferromagnetic Heisenberg model on the honeycomb lattice:

$$\tilde{H} = J \sum_{\langle \mathbf{r},\mathbf{r}' \rangle} \vec{S}_{\mathbf{r}} \cdot \vec{S}_{\mathbf{r}'} = J \sum_{\langle \mathbf{r},\mathbf{r}' \rangle} \left[S^z_{\mathbf{r}} S^z_{\mathbf{r}'} + \frac{1}{2} \left(S^+_{\mathbf{r}} S^-_{\mathbf{r}'} + S^-_{\mathbf{r}} S^+_{\mathbf{r}'} \right) \right]. \tag{5}$$

To calculate the dynamical spin structure factor in linear spin-wave theory, we represent the spin operators $\vec{S}_{\mathbf{r}}$ with standard bosonic operators via the Holstein-Primakoff transformation. Assuming an antiferromagnetic ordering at zero temperature, we can choose the spins in the A (B) sublattice to point in the $+z$ ($-z$) direction without loss of generality. The Holstein-Primakoff transformations on the two sublattices are then given by

$$S^z_{\mathbf{r}\in A} = \frac{1}{2} - a^\dagger_{A,\mathbf{r}} a_{A,\mathbf{r}}, \qquad S^+_{\mathbf{r}\in A} = \left(1 - a^\dagger_{A,\mathbf{r}} a_{A,\mathbf{r}}\right)^{1/2} a_{A,\mathbf{r}}, \qquad S^-_{\mathbf{r}\in A} = a^\dagger_{A,\mathbf{r}} \left(1 - a^\dagger_{A,\mathbf{r}} a_{A,\mathbf{r}}\right)^{1/2},$$

$$S^z_{\mathbf{r}\in B} = a^\dagger_{B,\mathbf{r}} a_{B,\mathbf{r}} - \frac{1}{2}, \qquad S^+_{\mathbf{r}\in B} = a^\dagger_{B,\mathbf{r}} \left(1 - a^\dagger_{B,\mathbf{r}} a_{B,\mathbf{r}}\right)^{1/2}, \qquad S^-_{\mathbf{r}\in B} = \left(1 - a^\dagger_{B,\mathbf{r}} a_{B,\mathbf{r}}\right)^{1/2} a_{B,\mathbf{r}}. \tag{6}$$

The linear spin-wave Hamiltonian is obtained by substituting Eq. (6) into Eq. (5) and expanding the result up to quadratic order in the bosonic operators $a^{(\dagger)}_{A,\mathbf{r}}$ and $a^{(\dagger)}_{B,\mathbf{r}}$. Following these steps, the linear spin-wave Hamiltonian takes the form

$$H = \frac{J}{2} \sum_{\mathbf{r}\in A} \sum_{\alpha=x,y,z} \left[-\frac{1}{2} + a^\dagger_{A,\mathbf{r}} a_{A,\mathbf{r}} + a^\dagger_{B,\mathbf{r}+\hat{\mathbf{r}}_\alpha} a_{B,\mathbf{r}+\hat{\mathbf{r}}_\alpha} + a_{A,\mathbf{r}} a_{B,\mathbf{r}+\hat{\mathbf{r}}_\alpha} + a^\dagger_{A,\mathbf{r}} a^\dagger_{B,\mathbf{r}+\hat{\mathbf{r}}_\alpha} \right], \tag{7}$$

where $\hat{\mathbf{r}}_{x,y,z}$ are the three vectors connecting any site in sublattice A to its three respective neighbors in sublattice B. Introducing momentum-space bosonic operators via the Fourier transforms

$$a_{A,\mathbf{r}} = \frac{1}{\sqrt{N}} \sum_{\mathbf{k}} \hat{a}_{A,\mathbf{k}}\, e^{i\mathbf{k}\cdot\mathbf{r}}, \qquad a_{B,\mathbf{r}} = \frac{1}{\sqrt{N}} \sum_{\mathbf{k}} \hat{a}_{B,\mathbf{k}}\, e^{i\mathbf{k}\cdot\mathbf{r}}, \tag{8}$$

where N is the number of honeycomb unit cells, the linear spin-wave Hamiltonian then becomes

$$\begin{aligned} H &= \frac{3J}{2} \sum_{\mathbf{k}} \left[-\frac{1}{2} + \hat{a}^\dagger_{A,\mathbf{k}} \hat{a}_{A,\mathbf{k}} + \hat{a}^\dagger_{B,\mathbf{k}} \hat{a}_{B,\mathbf{k}} + \lambda^*_{\mathbf{k}} \hat{a}_{A,\mathbf{k}} \hat{a}_{B,-\mathbf{k}} + \lambda_{\mathbf{k}} \hat{a}^\dagger_{A,\mathbf{k}} \hat{a}^\dagger_{B,-\mathbf{k}} \right] \\ &= -\frac{9JN}{4} + \frac{3J}{2} \sum_{\mathbf{k}} \begin{pmatrix} \hat{a}^\dagger_{A,\mathbf{k}} & \hat{a}_{B,-\mathbf{k}} \end{pmatrix} \begin{pmatrix} 1 & \lambda_{\mathbf{k}} \\ \lambda^*_{\mathbf{k}} & 1 \end{pmatrix} \begin{pmatrix} \hat{a}_{A,\mathbf{k}} \\ \hat{a}^\dagger_{B,-\mathbf{k}} \end{pmatrix}, \end{aligned} \tag{9}$$

where $\lambda_{\mathbf{k}} = \lambda^*_{-\mathbf{k}} = \frac{1}{3}\sum_\alpha e^{i\mathbf{k}\cdot\hat{\mathbf{r}}_\alpha}$. Finally, after an appropriate Bogoliubov transformation

$$\begin{pmatrix} \hat{a}_{A,\mathbf{k}} \\ \hat{a}^\dagger_{B,-\mathbf{k}} \end{pmatrix} = \begin{pmatrix} e^{i\vartheta_{\mathbf{k}}}\cosh\varphi_{\mathbf{k}} & e^{i\vartheta_{\mathbf{k}}}\sinh\varphi_{\mathbf{k}} \\ \sinh\varphi_{\mathbf{k}} & \cosh\varphi_{\mathbf{k}} \end{pmatrix} \begin{pmatrix} \hat{b}_{A,\mathbf{k}} \\ \hat{b}^\dagger_{B,-\mathbf{k}} \end{pmatrix}, \tag{10}$$

where $e^{i\vartheta_{\mathbf{k}}} = \lambda_{\mathbf{k}}/|\lambda_{\mathbf{k}}|$ and $\tanh 2\varphi_{\mathbf{k}} = -|\lambda_{\mathbf{k}}|$, the spin-wave Hamiltonian in Eq. (9) assumes the standard free-boson form

$$H = -\frac{9JN}{4} + \frac{3J}{2}\sum_{\mathbf{k}} \sqrt{1-|\lambda_{\mathbf{k}}|^2}\left[1 + \hat{b}^\dagger_{A,\mathbf{k}}\hat{b}_{A,\mathbf{k}} + \hat{b}^\dagger_{B,\mathbf{k}}\hat{b}_{B,\mathbf{k}}\right] \equiv E_0 + \sum_{\mathbf{k}} \varepsilon_{\mathbf{k}}\left[\hat{b}^\dagger_{A,\mathbf{k}}\hat{b}_{A,\mathbf{k}} + \hat{b}^\dagger_{B,\mathbf{k}}\hat{b}_{B,\mathbf{k}}\right], \tag{11}$$

where $\varepsilon_{\mathbf{k}} = \frac{3J}{2}\sqrt{1-|\lambda_{\mathbf{k}}|^2}$ is the spin-wave energy dispersion. Note that there are two degenerate spin waves $\hat{b}_{A,\mathbf{k}}$ and $\hat{b}_{B,\mathbf{k}}$ at each momentum $\mathbf{k}$ due to the sublattice structure of the honeycomb lattice.

The dynamical spin structure factor can be written as a sum of a transverse and a longitudinal contribution:

$$\mathcal{S}(\mathbf{q},\omega) = \mathcal{S}_\pm(\mathbf{q},\omega) + \mathcal{S}_{zz}(\mathbf{q},\omega). \tag{12}$$

In linear spin-wave theory, the transverse contribution is given by

$$\mathcal{S}_\pm(\mathbf{q},\omega) = \frac{1}{4\pi N}\sum_{\mathbf{r},\mathbf{r}'}\int_{-\infty}^{+\infty} dt\, e^{i\omega t - i\mathbf{q}\cdot(\mathbf{r}'-\mathbf{r})}\Big[g_x^2\langle 0|S^x_{\mathbf{r}'}(t)S^x_{\mathbf{r}}(0)|0\rangle + g_y^2\langle 0|S^y_{\mathbf{r}'}(t)S^y_{\mathbf{r}}(0)|0\rangle\Big], \tag{13}$$

where $g_{x,y}$ are appropriate g factors within the (xy) plane, $|0\rangle$ is the ground state of the spin-wave Hamiltonian H [see Eq. (11)], defined by $\hat{b}_{A,\mathbf{k}}|0\rangle = \hat{b}_{B,\mathbf{k}}|0\rangle = 0$ for all $\mathbf{k}$, and the time-evolved spin operators are approximated as

$$\begin{aligned} S^x_{\mathbf{r}\in A}(t) &= \frac{1}{2}e^{iHt}\left(a_{A,\mathbf{r}} + a^\dagger_{A,\mathbf{r}}\right)e^{-iHt}, & S^y_{\mathbf{r}\in A}(t) &= -\frac{i}{2}e^{iHt}\left(a_{A,\mathbf{r}} - a^\dagger_{A,\mathbf{r}}\right)e^{-iHt}, \\ S^x_{\mathbf{r}\in B}(t) &= \frac{1}{2}e^{iHt}\left(a_{B,\mathbf{r}} + a^\dagger_{B,\mathbf{r}}\right)e^{-iHt}, & S^y_{\mathbf{r}\in B}(t) &= \frac{i}{2}e^{iHt}\left(a_{B,\mathbf{r}} - a^\dagger_{B,\mathbf{r}}\right)e^{-iHt}. \end{aligned} \tag{14}$$

Substituting Eq. (14) into Eq. (13), and using Eqs. (8) and (10), the transverse contribution then becomes

$$\mathcal{S}_\pm(\mathbf{q},\omega) = \frac{(g_x^2+g_y^2)(1-|\lambda_{\mathbf{q}}|\cos\vartheta_{\mathbf{q}})}{4\sqrt{1-|\lambda_{\mathbf{q}}|^2}}\,\delta\left(\omega-\varepsilon_{\mathbf{q}}\right). \tag{15}$$

This contribution gives a sharp feature in the dynamical spin structure factor, which directly corresponds to the spin-wave energy dispersion $\varepsilon_{\mathbf{q}}$. In contrast, the longitudinal contribution is given by

$$\mathcal{S}_{zz}(\mathbf{q},\omega) = \frac{g_z^2}{4\pi N}\sum_{\mathbf{r},\mathbf{r}'}\int_{-\infty}^{+\infty} dt\, e^{i\omega t - i\mathbf{q}\cdot(\mathbf{r}'-\mathbf{r})}\langle 0|S^z_{\mathbf{r}'}(t)S^z_{\mathbf{r}}(0)|0\rangle, \tag{16}$$

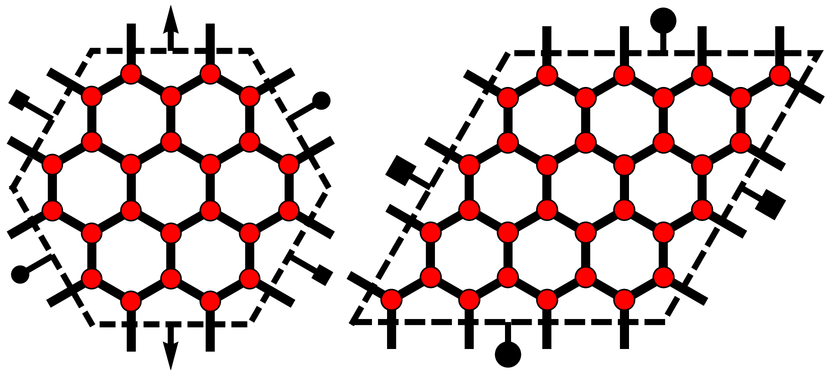

FIG. 8. Finite size clusters used for TPQ calculations. Left: 24-site cluster with periodic boundary conditions and C_3 symmetry. Edges with same symbols are identified. Right: 32-site rhombic cluster with periodic boundary conditions.

where g_z is the g factor in the z direction, and the time-evolved spin operators are

$$S^z_{\mathbf{r}\in A}(t) = e^{iHt}\left(\frac{1}{2} - a^\dagger_{A,\mathbf{r}} a_{A,\mathbf{r}}\right) e^{-iHt}, \qquad S^z_{\mathbf{r}\in B}(t) = e^{iHt}\left(a^\dagger_{B,\mathbf{r}} a_{B,\mathbf{r}} - \frac{1}{2}\right) e^{-iHt}. \tag{17}$$

Substituting Eq. (17) into Eq. (16), and ignoring any elastic contribution at $\omega = 0$, the longitudinal contribution takes the form

$$\mathcal{S}_{zz}(\mathbf{q},\omega) = \frac{g_z^2}{4N}\sum_{\mathbf{k}} \frac{1 - \sqrt{1-|\lambda_{\mathbf{k}}|^2}\sqrt{1-|\lambda_{\mathbf{q-k}}|^2} - |\lambda_{\mathbf{k}}||\lambda_{\mathbf{q-k}}|\cos\left(\vartheta_{\mathbf{k}} + \vartheta_{\mathbf{q-k}}\right)}{\sqrt{1-|\lambda_{\mathbf{k}}|^2}\sqrt{1-|\lambda_{\mathbf{q-k}}|^2}}\,\delta\left(\omega - \varepsilon_{\mathbf{k}} - \varepsilon_{\mathbf{q-k}}\right). \tag{18}$$

Because of the summation over the momentum $\mathbf{k}$, this contribution gives a broad continuum over an entire energy range for each momentum $\mathbf{q}$.

VI. NOTES ON THE MAGNETIC SPECIFIC HEAT CALCULATION

We numerically calculate C_{mag} of the AFM Heisenberg model on C_3-symmetric 24-site and rhombic 32-site clusters (see Fig. 8) with periodic boundary conditions using the microcanonical thermal pure quantum state (mTPQ) method [10] as implemented in the $\mathcal{H}\Phi$ library [11]. In the mTPQ approach, a quantum state at thermal equilibrium is approximated by a single state, iteratively generated from a random initial vector representing infinite temperature. At each iteration, the TPQ state is associated with a temperature estimated from the internal energy of the state, [10, 12] and used to calculate C_{mag}. We average over 15 initial vectors to reduce statistical error. Like the finite-size error, this mainly affects the lowest temperatures, as shown in Fig. 9. The lower

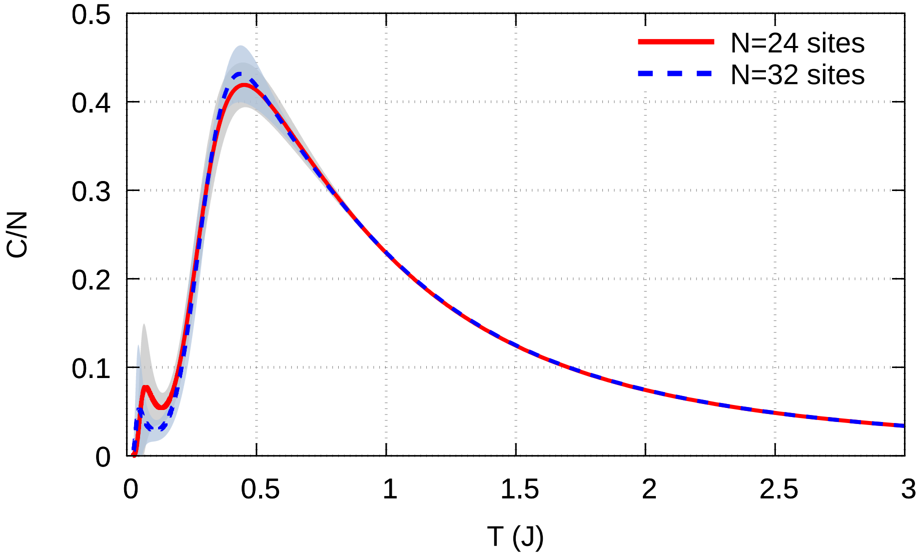

FIG. 9. Magnetic specific heat of the AFM Heisenberg model calculated using mTPQ and normalized by number of sites, for both the 24- and 32-site clusters. The lines show the average values, and shaded regions indicate the standard deviations. The finite-size effect is found to be weak, except at the lowest temperatures.

peak, which gets suppressed in temperature and magnitude with increasing system size, is likely a spurious result caused by the sparse low-energy spectrum of finite-size systems. For both cluster sizes it occurs at temperatures $T < 0.1J < 0.5$ K (using $J = 0.42$ meV), and thus outside the range of the experimental heat capacity measurements and fits discussed in the main text. Our numerical results are consistent with Ref. [12].

---

[1] May, A. F., Yan, J. & McGuire, M. A. A practical guide for crystal growth of van der Waals layered materials. *Journal of Applied Physics* **128**, 051101 (2020).

[2] Copley, J. & Cook, J. The disk chopper spectrometer at NIST: a new instrument for quasielastic neutron scattering studies. *Chemical Physics* **292**, 477 – 485 (2003). Quasielastic Neutron Scattering of Structural Dynamics in Condensed Matter.

[3] Ehlers, G., Podlesnyak, A. A., Niedziela, J. L., Iverson, E. B. & Sokol, P. E. The new cold neutron chopper spectrometer at the spallation neutron source: Design and performance. *Review of Scientific Instruments* **82**, 085108 (2011). URL `https://doi.org/10.1063/1.3626935`.

[4] Arnold, O. *et al.* Mantidâdata analysis and visualization package for neutron scattering and μSR experiments. *Nuclear Instruments and Methods in Physics Research Section A: Accelerators, Spectrometers, Detectors and Associated Equipment* **764**, 156 – 166 (2014).

[5] Winn, Barry *et al.* Recent progress on HYSPEC, and its polarization analysis capabilities. *EPJ Web of Conferences* **83**, 03017 (2015).

[6] Zaliznyak, I. A. *et al.* Polarized neutron scattering on HYSPEC: the HYbrid SPECtrometer at SNS. *Journal of Physics: Conference Series* **862**, 012030 (2017).

[7] Moon, R. M., Riste, T. & Koehler, W. C. Polarization analysis of thermal-neutron scattering. *Phys. Rev.* **181**, 920–931 (1969).

[8] Aroyo, M. *et al.* Crystallography online: Bilbao crystallographic server. *Bulgarian Chemical Communications* **43**, 183–197 (2011).

[9] Xing, J. *et al.* Néel-type antiferromagnetic order and magnetic field–temperature phase diagram in the spin-$\frac{1}{2}$ rare-earth honeycomb compound $YbCl_3$. *Phys. Rev. B* **102**, 014427 (2020).

[10] Sugiura, S. & Shimizu, A. Thermal pure quantum states at finite temperature. *Phys. Rev. Lett.* **108**, 240401 (2012).

[11] Kawamura, M. *et al.* Quantum lattice model solver $\mathcal{H}\Phi$. *Comp. Phys. Comms.* **217**, 180 (2017).

[12] Yamaji, Y. *et al.* Clues and criteria for designing a Kitaev spin liquid revealed by thermal and spin excitations of the honeycomb iridate Na_2IrO_3. *Phys. Rev. B* **93**, 174425 (2016).
