# Supplementary material for: Van Hove singularity in the magnon spectrum of the antiferromagnetic quantum honeycomb lattice
Source: Nat Commun. 2021 Jan 8;12:171. doi: 10.1038/s41467-020-20335-5 (PMC7794317; doi:10.1038/s41467-020-20335-5)
Supplement: Supplementary file 2 — Peer Review File [file 41467_2020_20335_MOESM2_ESM.pdf]

Editorial Note: This manuscript has been previously reviewed at another journal that is not operating a transparent peer review scheme. This document only contains reviewer comments and rebuttal letters for versions considered at *Nature Communications*. Mentions of prior referee reports have been redacted.
